# PINK1/Parkin-mediated mitophagy inhibits osteoblast apoptosis induced by advanced oxidation protein products

**DOI:** 10.1038/s41419-023-05595-5

**Published:** 2023-02-07

**Authors:** Wei Li, Wang-Sheng Jiang, Ya-Ru Su, Ke-Wu Tu, Lin Zou, Cong-Rui Liao, Qian Wu, Zi-Han Wang, Zhao-Ming Zhong, Jian-Ting Chen, Si-Yuan Zhu

**Affiliations:** 1 Division of Spine Surgery, Department of Orthopedics, Nanfang Hospital, Southern Medical University, Guangzhou, China; 2 Department of Ophthalmology, Nanfang Hospital, Southern Medical University, Guangzhou, China; 3 Department of Orthopedics, Nanfang Hospital Taihe Branch, Southern Medical University, Guangzhou, China

**Keywords:** Mitophagy, Calcium and phosphate metabolic disorders

## Abstract

Osteoblast apoptosis plays an important role in age-related bone loss and osteoporosis. Our previous study revealed that advanced oxidation protein products (AOPPs) could induce nicotinamide adenine dinucleotide phosphate oxidase (NOX)-derived reactive oxygen species (ROS) production, cause mitochondrial membrane potential (ΔΨm) depolarization, trigger the mitochondria-dependent intrinsic apoptosis pathway, and lead to osteoblast apoptosis and ultimately osteopenia and bone microstructural destruction. In this study, we found that AOPPs also induced mitochondrial ROS (mtROS) generation in osteoblastic MC3T3-E1 cells, which was closely related to NOX-derived ROS, and aggravated the oxidative stress condition, thereby further promoting apoptosis. Removing excessive ROS and damaged mitochondria is the key factor in reversing AOPP-induced apoptosis. Here, by in vitro studies, we showed that rapamycin further activated PINK1/Parkin-mediated mitophagy in AOPP-stimulated MC3T3-E1 cells and significantly alleviated AOPP-induced cell apoptosis by eliminating ROS and damaged mitochondria. Our in vivo studies revealed that PINK1/Parkin-mediated mitophagy could decrease the plasma AOPP concentration and inhibit AOPP-induced osteoblast apoptosis, thus ameliorating AOPP accumulation-related bone loss, bone microstructural destruction and bone mineral density (BMD) loss. Together, our study indicated that therapeutic strategies aimed at upregulating osteoblast mitophagy and preserving mitochondrial function might have potential for treating age-related osteoporosis.

## Introduction

Advanced oxidation protein products (AOPPs) are considered a biomarker of oxidative damage to proteins [1–3], which has been reported in the aging process [4] and the development of some age-related diseases [5–7]. Our group and others have reported that AOPPs are a marker of oxidative stress in bone [5, 6, 8, 9]. The AOPP content in plasma or bone tissue increases with aging and has a negative relationship with bone mass [4, 5, 10]. We previously reported that AOPPs could activate nicotinamide adenine dinucleotide phosphate (NADPH) oxidases (NOXs), produce massive amounts of reactive oxygen species (ROS), and induce oxidative stress in osteoblasts, thereby causing mitochondrial membrane potential (ΔΨm) depolarization and triggering the mitochondria-dependent intrinsic apoptosis pathway [5]. Thus, AOPP-induced osteoblast apoptosis played an important role in age-related bone loss. However, in addition to NOX-derived ROS, mitochondrial ROS (mtROS) are the primary source of intracellular ROS [11]. In normal circumstances, mtROS are isolated inside mitochondria, but when mitochondria are damaged, mtROS will be released into the cytoplasm, where they further aggravate cellular oxidative stress [12]. Based on the ROS-induced ROS release (RIRR) mechanism, NOX-derived ROS may also induce mtROS production, thereby worsening mitochondrial damage and further promoting cell apoptosis [13, 14]. Therefore, it is important to determine whether AOPPs can induce mtROS generation and whether eliminating mtROS or damaged mitochondria alleviates AOPP-induced osteoblast apoptosis, consequently alleviating AOPP-induced osteopenia.

It is known that damaged mitochondria and mtROS can be cleared by mitophagy, a highly conserved selective autophagy process that maintains mitochondrial quality and quantity [15]. Through mitophagy, cells can cope with mitochondrial stress or oxidative stress and keep damage manageable, which leads to cell survival [16]. Increasing evidence has demonstrated that mitophagy can promote osteoblast survival, increase bone volume, and alleviate osteoporosis [17–19]. Currently, the PTEN-induced kinase 1 (PINK1)/Parkin-mediated ubiquitin-driven mitophagy has been well studied in mammals under conditions of ΔΨm loss [20, 21]. PINK1 is a mitochondrial serine-threonine kinase that is stabilized on the outer mitochondrial membrane and senses the mitochondrial health status [21, 22]. Upon ΔΨm depolarization, PINK1 recruits Parkin, a cytosolic E3 ubiquitin ligase, to mitochondria and activates mitophagy [21, 22].

In this study, the relationships between AOPPs, mtROS, PINK1/Parkin-mediated mitophagy and osteoblast apoptosis were explored in depth. We found that AOPP accumulation induced mtROS overproduction in osteoblasts, while PINK1/Parkin-mediated mitophagy eliminated damaged or dysfunctional mitochondria, decreased excessive intracellular ROS production, and inhibited osteoblast apoptosis, thus relieving the bone loss induced by AOPPs.

## Results

### AOPPs induced mitochondrial ROS (mtROS) production in MC3T3-E1 cells

**Fig. 1:**
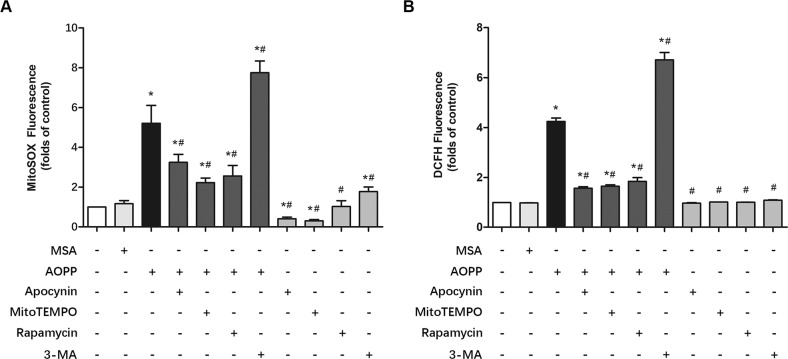
AOPPs induced mtROS production in MC3T3-E1 cells. **A** MitoSOX results showed that AOPPs (200 μg/mL) significantly increased mtROS levels in MC3T3-E1 cells. Pretreatment with apocynin, MitoTEMPO or rapamycin significantly reduced AOPP-induced mtROS production. Apocynin or MitoTEMPO alone also decreased mtROS levels in MC3T3-E1 cells, while rapamycin alone had no significant effect on mtROS levels. However, 3-MA not only elevated the mtROS level when administered alone but also further increased AOPP-induced mtROS generation (*n* = 6 for each group). **B** DCFH-DA results showed that the intracellular ROS production induced by 200 μg/mL AOPPs could be decreased by pretreatment with apocynin, MitoTEMPO and rapamycin. 3-MA further increased the intracellular ROS generation induced by AOPPs. Apocynin, MitoTEMPO, rapamycin and 3-MA had no significant effect on intracellular ROS production compared to the control group (*n* = 6 for each group). Data are presented as the mean ± SD. *P < 0.05 versus control, ^#^*P* < 0.05 versus AOPPs.

The mitochondrial superoxide indicator MitoSOX was applied to determine whether AOPPs could induce mtROS production in MC3T3-E1 cells. As shown in Fig. 1A, AOPPs (200 μg/ml, incubated for 24 h) significantly increased mtROS production in MC3T3-E1 cells, while apocynin, an NOX inhibitor, markedly decreased AOPP-induced mtROS production, which indicated that NOX-derived ROS may influence mtROS release. Moreover, both the specific mtROS scavenger MitoTEMPO and the mitophagy promotor rapamycin significantly decreased mtROS levels. Furthermore, we found that the mitophagy inhibitor 3-MA dramatically increased mtROS generation. We next used 2’,7’-dichlorofluorescein diacetate (DCFH-DA) to detect the total intracellular ROS production. A similar result was observed in Fig. 1B. Briefly, AOPPs (200 μg/ml, incubated for 24 h) increased total ROS production, whereas apocynin, MitoTEMPO and rapamycin significantly decreased AOPP-induced ROS production. In contrast, 3-MA further elevated ROS levels. Notably, we also found that MitoTEMPO, rapamycin, and 3-MA alone did not influence total ROS production in MC3T3-E1 cells (Fig. 1B). However, the MitoSOX results showed that apocynin and MitoTEMPO further decreased mtROS compared with the blank control, while 3-MA still increased mtROS generation (Fig. 1A).

### AOPPs induced mitophagy in MCET3-E1 cells

**Fig. 2:**
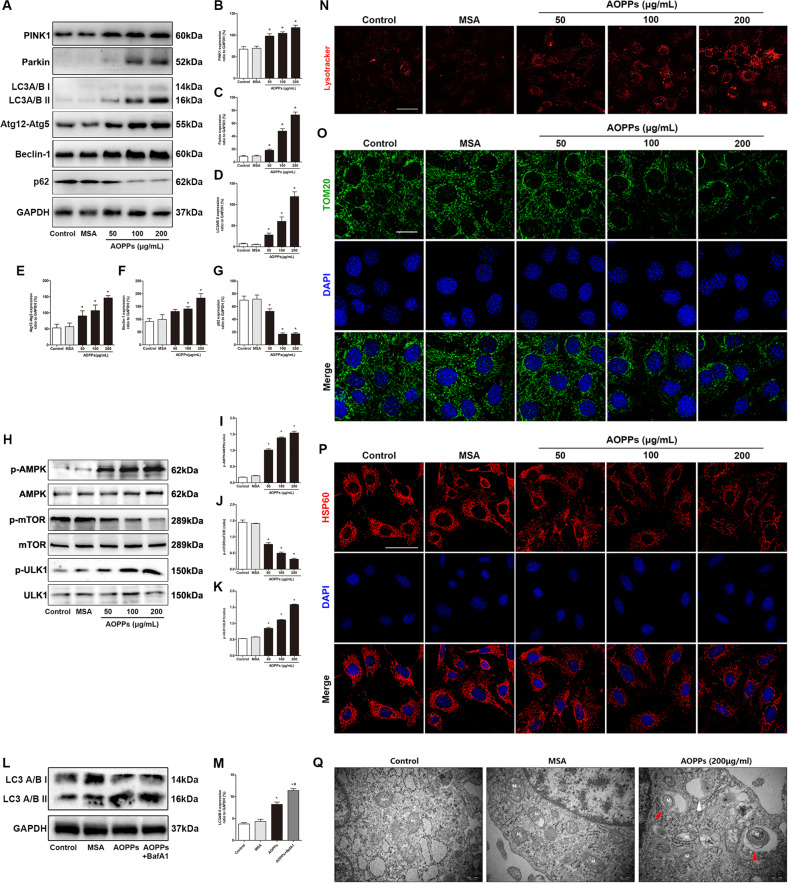
AOPPs induced mitophagy in MCET3-E1 cells. **A**–**G** AOPP treatment significantly increased PINK1, Parkin, LC3A/B-II, Atg12-Atg5, and Beclin-1 expression while decreasing p62 expression in a concentration-dependent manner. **H**–**K** AOPP treatment significantly increased the phosphorylation of AMPK and ULK1 but inhibited the phosphorylation of mTOR in a concentration-dependent manner. **L**, **M** The expression level of LC3A/B-II in AOPPs+BafA1 was significantly higher than AOPPs group. **N** Representative confocal microscope images using LysoTracker Red revealed that AOPPs induced lysosome generation in MC3T3-E1 cells in a concentration-dependent manner (scale bar = 20 μm). **O** Representative confocal microscope images showed that AOPPs induced TOM20 degradation in a concentration-dependent manner (scale bar = 20 μm). **P** Representative confocal microscope images showed that AOPPs induced HSP60 degradation in a concentration-dependent manner (scale bar = 50 μm). **Q** Representative TEM images showing autophagosomes (white arrow) and mitophagy (red arrow, M: mitochondria) in MC3T3-E1 cells after AOPP treatment (scale bar = 500 nm). All experiments were repeated for at least three times. Data are presented as the mean ± SD. *P < 0.05 versus control.

We next incubated cells with increasing concentrations (0–200 μg/ml) of AOPPs for 24 h, and found that the administration of AOPPs significantly increased the expression of PINK1, Parkin, LC3A/B-II, Atg12-Atg5, Beclin-1, p-AMPK/AMPK, and p-ULK1/ULK1 while decreasing p62 and p-mTOR/mTOR expression in a dose-dependent manner (Fig. 2A–K). Autophagosome-lysosome fusion is crucial for autophagic flux. Bafilomycin A1 is a mitophagosome-lysosome fusion inhibitor, as shown in Fig. 2L, M, the expression level of LC3A/B-II in AOPPs+Bafilomycin A1 group was significantly higher than AOPPs group, which revealed the enhanced mitophagy flux in AOPP-stimulated cells. The LysoTracker Red results also showed marked production of lysosomes accompanied by increased AOPP concentrations (Fig. 2N). Moreover, immunofluorescent staining showed that the administration of AOPPs induced prominent degradation of the mitochondrial outer membrane protein TOM20 and mitochondrial matrix protein HSP60 in MC3T3-E1 cells, which also indicated mitophagy in AOPP-treated MC3T3-E1 cells (Fig. 2O, P). Furthermore, the transmission electron microscopy (TEM) results provided direct evidence of mitophagy, and isolation membranes were observed to form around mitochondria in MCET3-E1 cells after 24 h of stimulation with 200 μg/mL AOPPs (Fig. 2Q).

Additionally, the expression of Optineurin (OPTN), a xenophagy indicator, showed no significant difference among each group during AOPPs stimulation (0–200 μg/ml, 24 h) in MC3T3-E1 cells, which was shown in Supplementary Material Fig. 1.

### PINK1/Parkin-mediated mitophagy inhibited AOPP-induced MCET3-E1 cell apoptosis

**Fig. 3:**
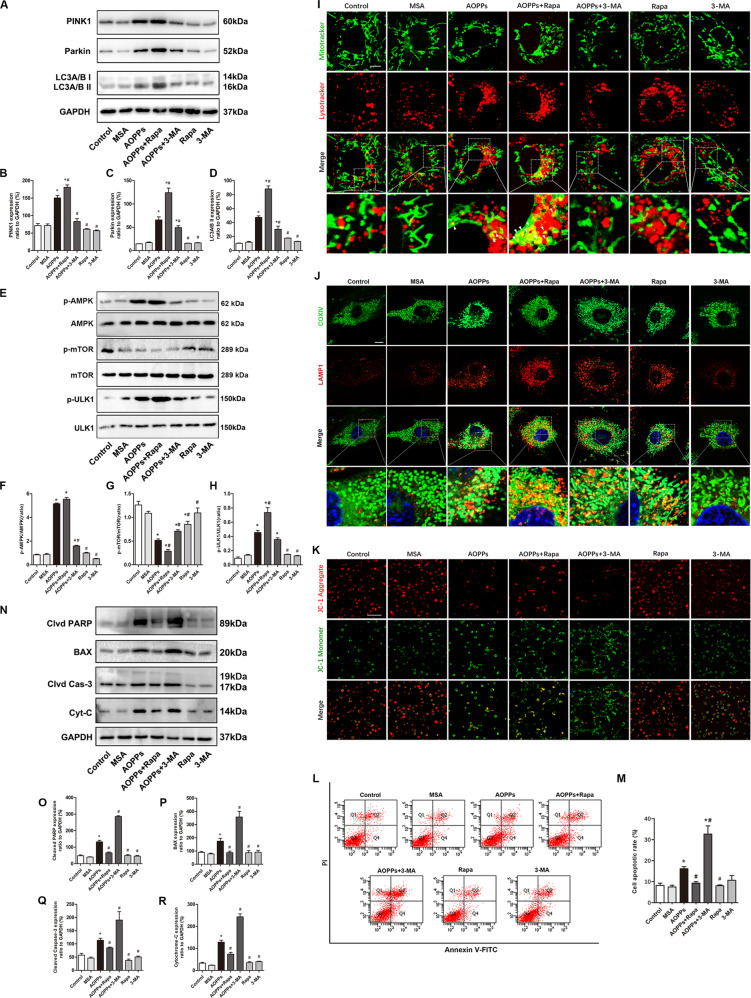
PINK1/Parkin-mediated mitophagy inhibited AOPP-induced MC3T3-E1 cell apoptosis. **A**–**D** AOPP treatment significantly increased PINK1, Parkin, and LC3A/B-II expression, and this tendency was further upregulated by rapamycin or inhibited by 3-MA. **E**–**H** AOPPs increased the phosphorylation of AMPK and ULK1 while decreasing mTOR phosphorylation. Rapamycin decreased the phosphorylation of mTOR but had no significant effect on AMPK or ULK1 phosphorylation. Rapamycin combined with AOPPs further decreased the phosphorylation of mTOR and increased the phosphorylation ULK1, but had no significant effect on AMPK phosphorylation. 3-MA significantly decreased the AOPP-induced phosphorylation of AMPK and ULK1. **I** The MitoTracker Green/LysoTracker Red fluorescence results showed that AOPP stimulation significantly increased mitophagy (white arrow), and this tendency could be further enhanced by rapamycin or inhibited by 3-MA (scale bar = 10 μm). **J** Co-localization of mitochondrial protein COX IV and LAMP1 by immunofluorescence staining showed AOPP stimulation significantly increased mitophagy, and this tendency could be further enhanced by rapamycin or inhibited by 3-MA (scale bar = 10 μm). **K** The JC-1 results showed that rapamycin could significantly reverse the AOPP-induced ΔΨm decrease, but 3-MA further exacerbated the damaging effect of AOPPs on mitochondria (scale bar = 100 μm). **L**, **M** Flow cytometry results showed that AOPP-induced MC3T3-E1 cell apoptosis could be inhibited by rapamycin but further exacerbated by 3-MA. Rapamycin or 3-MA alone had no significant effect on the cell apoptotic rate. **N**–**R** The AOPP-induced expression of cleaved PARP, BAX, cleaved Caspase-3 and cytochrome c was significantly inhibited by rapamycin but further upregulated by 3-MA. However, rapamycin or 3-MA alone had no significant effect on apoptotic protein expression compared with the control group. All experiments were repeated for at least three times. Data are presented as the mean ± SD. *P < 0.05 versus control. ^#^*P* < 0.05 versus the AOPPs group. Clvd PARP, cleaved PARP; Clvd Cas-3 cleaved Caspase-3; Cyt-C cytochrome c.

To further investigate the effect of mitophagy on the survival of AOPP-treated (200 μg/ml, incubated for 24 h) MC3T3E1 cells, rapamycin and 3-MA were applied to upregulate or downregulate mitophagy levels, respectively. AOPP treatment significantly increased PINK1, Parkin, and LC3A/B-II expression, and the tendency could be further upregulated by rapamycin or inhibited by 3-MA (Fig. 3A–D). The activation state of the signaling pathway AMPK/mTOR/ULK1 upstream of autophagy was also detected. As shown in Fig. 3E–H, AOPPs increased the phosphorylation of AMPK and ULK1 while decreasing mTOR phosphorylation. Interestingly, rapamycin decreased the phosphorylation of mTOR but had no significant effect on AMPK phosphorylation. In addition, we found that rapamycin combined with AOPPs further decreased the phosphorylation of mTOR and increased the phosphorylation of ULK1 but had no significant effect on AMPK phosphorylation compared with that in the AOPPs group. However, 3-MA significantly decreased the AOPP-induced phosphorylation of AMPK and ULK1.

Costaining with MitoTracker Green and LysoTracker Red was used to locate mitophagy events in MC3T3-E1 cells. AOPP stimulation (200 μg/ml, incubated for 24 h) significantly increased mitophagy, and this tendency was further enhanced by rapamycin or inhibited by 3-MA. In addition, neither rapamycin alone nor 3-MA alone had a significant effect on mitophagy in normal MC3T3-E1 cells (Fig. 3I). We also conducted co-localization of mitochondrial protein COX IV and lysosomal associated membrane protein 1 (LAMP1) by immunofluorescence staining, which also showed a similar tendency (Fig. 3J). JC-1 staining was used to detect changes in mitochondrial membrane potential. As shown in Fig. 3K, rapamycin significantly reversed the AOPP-induced decrease in ΔΨm, but 3-MA further exacerbated the damaging effect of AOPPs on mitochondria. However, neither rapamycin alone nor 3-MA alone had a significant effect on the ΔΨm of MC3T3-E1 cells in our study.

**Fig. 4:**
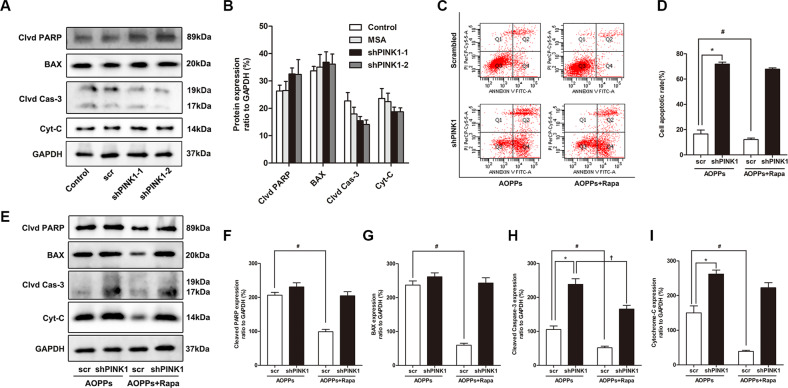
PINK1 knockdown aggravated AOPP-induced osteoblast apoptosis. **A**, **B** PINK1 knockdown by shRNA had no significant effect on the expression of cleaved PARP, BAX, cleaved Caspase-3, or cytochrome c in MC3T3-E1 cells. **C**, **D** Flow cytometry results showed that PINK1 knockdown further increased the cell apoptotic rate when stimulated with AOPPs, and the application of rapamycin did not reverse this trend. **E**–**I** Rapamycin failed to inhibit the upregulation of cleaved PARP, BAX and cytochrome c in AOPP-stimulated shPINK1 MC3T3-E1 cells. All experiments were repeated for at least three times. Data are presented as the mean ± SD. *^#†^*P* < 0.05 between connected groups. Clvd PARP cleaved PARP; Clvd Cas-3 cleaved Caspase-3; Cyt-C cytochrome c.

The effect of mitophagy on AOPP-induced (200 μg/ml, incubated for 24 h) cell death was detected. Flow cytometry assays using Annexin V-fluorescein isothiocyanate (FITC)/propidium iodide (PI) staining showed that rapamycin attenuated AOPP-induced cell apoptosis and that costimulation with 3-MA and AOPPs induced an even higher apoptosis rate than AOPP treatment alone (Fig. 3L, M). As shown in Fig. 3N–R, rapamycin pretreatment decreased the expression of the apoptosis-related proteins cleaved PARP, BAX, cleaved Caspase-3 and cytochrome c in AOPP-stimulated MC3T3-E1 cells, while 3-MA further increased the protein expression. To further clarify the role of the PINK1/Parkin-mediated mitophagy process in AOPP-induced osteoblast apoptosis, we knocked down PINK1 with shRNA in MC3T3-E1 cells. As shown in Fig. 4A, B, PINK1 knockdown alone had no influence on the baseline expression levels of cleaved PARP, BAX, cleaved Caspase-3 and cytochrome c in MC3T3-E1 cells. The flow cytometry analysis results (Fig. 4C, D) showed that when treated with AOPPs, the shPINK1 group had a dramatically higher apoptotic rate than the scrambled group, and rapamycin failed to rescue these PINK1 knockdown cells from apoptosis. Similar to the flow cytometry results, rapamycin failed to inhibit the AOPP-induced expression of cleaved PARP, BAX and cytochrome c in the shPINK1 group but still inhibited their expression in the scrambled group (Fig. 4E–I). Together, these results indicated that PINK1/Parkin-mediated mitophagy played a self-protective role in preventing AOPP-induced apoptosis of osteoblasts.

### PINK1/Parkin-mediated mitophagy inhibited AOPP-induced osteoblast apoptosis in vivo

**Fig. 5:**
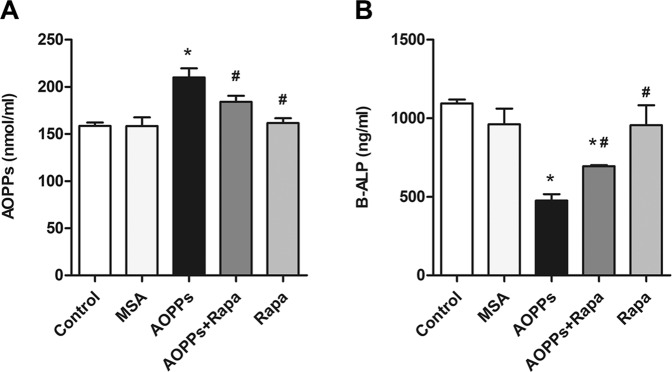
Plasma AOPP and B-ALP concentrations in each group. **A** The intraperitoneal injection of AOPPs daily for 16 weeks markedly increased the plasma AOPP level, while rapamycin intervention significantly inhibited this trend. **B** The intraperitoneal injection of AOPPs daily for 16 weeks markedly decreased the plasma B-ALP level, while rapamycin intervention significantly inhibited this trend. Data are presented as the mean ± SD, *P < 0.05 versus control, ^#^*P* < 0.05 versus AOPPs, *n* = 8.

**Fig. 6:**
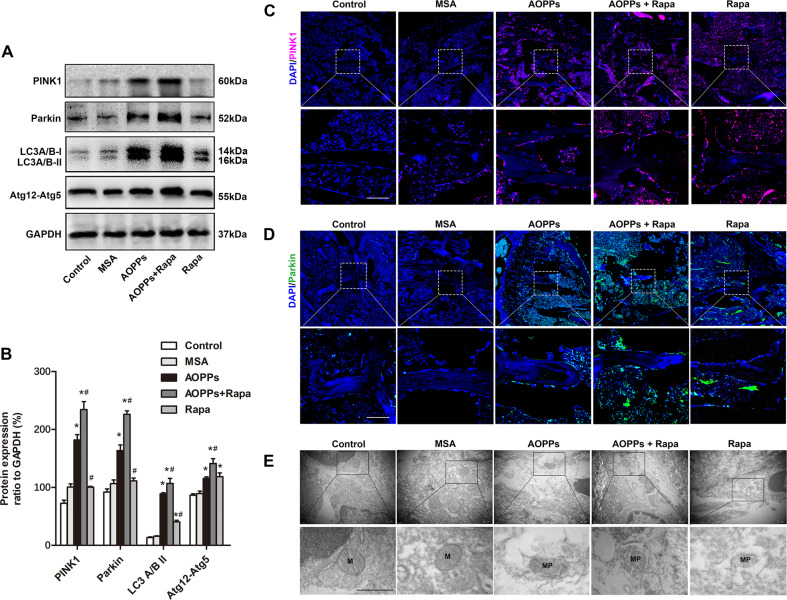
Rapamycin further upregulated PINK1/Parkin-mediated mitophagy in AOPP-stimulated mice. **A**, **B** Western blot analysis showed that AOPP accumulation upregulated the expression of PINK1, Parkin, LC3A/B-II and Atg12-Atg5 in tibial bone tissues, which was further upregulated by additional rapamycin treatment. **C**, **D** Immunofluorescent staining results showed that both AOPPs and rapamycin could increase the expression of PINK1 and Parkin in osteoblasts, while the highest expression level was observed in the AOPPs+Rapamycin group (scale bar = 50 μm). **E** Representative TEM images showing mitophagy (marked as MP) in the AOPPs, AOPPs+Rapamycin, and Rapamycin groups (scale bar = 500 nm). Data are presented as the mean ± SD, *P < 0.05 versus control, ^#^*P* < 0.05 versus AOPPs, *n* = 8.

The intraperitoneal injection of AOPPs (50 mg/kg) daily for 16 weeks markedly increased the plasma AOPP levels while decreasing bone-specific alkaline phosphatase (B-ALP) levels, while rapamycin intervention significantly increased plasma B-ALP levels and decreased AOPP levels (Fig. 5A, B). Western blot analysis of tibia bone tissue showed that the expression levels of the mitophagy-related proteins PINK1, Parkin, LC3A/B-II and Atg12-Atg5 were significantly upregulated in the AOPPs group, the Rapamycin group and the AOPPs+Rapamycin group. The AOPPs+Rapamycin group showed the highest protein expression level among the five groups (Fig. 6A, B). The tibia immunofluorescence results of PINK1 and Parkin showed the same tendency (Fig. 6C, D). Moreover, the mitophagy of osteoblasts in tibia bone tissue was directly observed by TEM, which further confirmed our results (Fig. 6E). Together, these results indicated that PINK1/Parkin-mediated mitophagy was activated in mice with AOPP accumulation and could be further promoted by rapamycin.

**Fig. 7:**
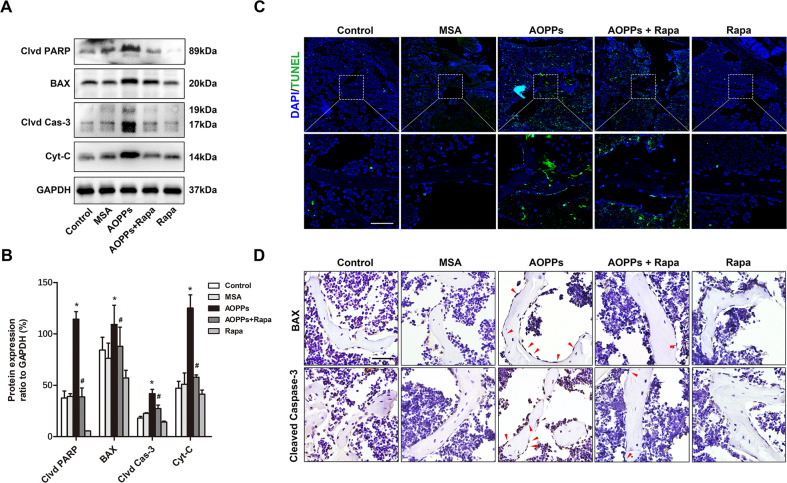
Mitophagy inhibited AOPP-induced apoptosis of osteoblast in mice. **A**, **B** Western blot results of the tibia bone tissue showed that rapamycin significantly decreased the AOPP-induced upregulation of cleaved PARP, BAX, cleaved Caspase-3, and cytochrome c. **C** TUNEL assay results showed that rapamycin significantly inhibited AOPP-induced osteoblast apoptosis in vivo (scale bar = 50 μm). **D** Immunohistochemical results showed that rapamycin inhibited the AOPP-induced expression of BAX and cleaved Caspase-3 in osteoblasts in vivo (scale bar = 50 μm). Data are presented as the mean ± SD, *P < 0.05 versus control, ^#^*P* < 0.05 versus AOPPs, *n* = 8. Clvd PARP cleaved PARP; Clvd Cas-3 cleaved Caspase-3; Cyt-C cytochrome c.

Moreover, the expression levels of the apoptosis-related proteins cleaved PARP, BAX, cleaved Caspase-3, and cytochrome c were detected in each group. As shown in Fig. 7A, B, AOPP stimulation significantly increased the protein expression level, and this effect could be inhibited by rapamycin, which was consistent with our in vitro results. In addition, both TUNEL staining of apoptotic osteoblasts (Fig. 7C) and immunohistochemical staining of BAX and cleaved Caspase-3 showed similar results (Fig. 7D).

### PINK1/Parkin-mediated mitophagy inhibited AOPP-induced bone loss and bone microstructural destruction

**Fig. 8:**
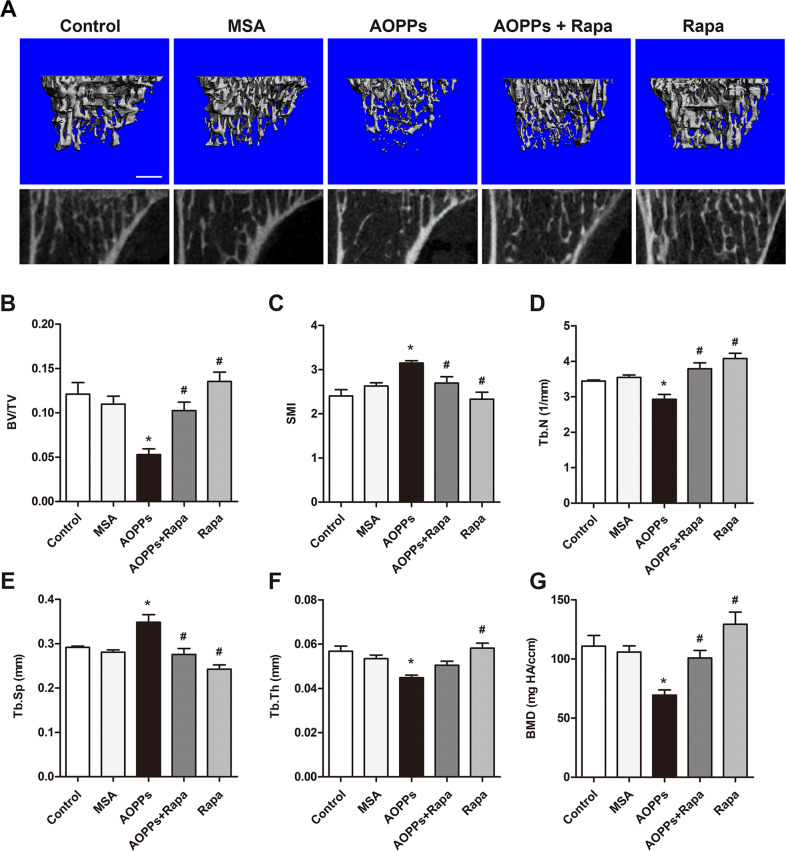
Mitophagy ameliorated osteopenia and bone microstructural destruction induced by AOPP accumulation. **A**–**G** Micro-CT evaluation revealed that chronic AOPP loading significantly decreased BV/TV, Tb. N, and Tb. Th, while increasing Tb. Sp and SMI in the proximal tibias of mice. This finding was further confirmed by representative three-dimensional reconstructions (Scale bar = 500 μm). In addition, we also found that AOPP stimulation decreased the bone mineral density (BMD) in the trabeculae of the proximal tibias. Notably, rapamycin alleviated the deterioration of bone microstructure and the decrease in BMD caused by AOPP treatment. Data are presented as the mean ± SD, *P < 0.05 versus control, ^#^*P* < 0.05 versus AOPPs, *n* = 8.

As shown in Fig. 8A–F, micro-CT evaluation revealed that chronic AOPP loading significantly decreased bone volume over total volume (BV/TV), trabecular number (Tb. N), and trabecular thickness (Tb. Th), while increasing the trabecular spacing (Tb. Sp) and structure model index (SMI) in the proximal tibias of mice. This finding was further confirmed by representative three-dimensional reconstructions. In addition, we also found that AOPP stimulation decreased the bone mineral density (BMD) in the trabeculae of the proximal tibias (Fig. 8G). Notably, the administration of rapamycin alleviated the deterioration of bone microstructure and the decrease in BMD caused by AOPP treatment.

## Discussion

AOPPs are dityrosine-containing cross-linked proteins that arise from the reaction between blood proteins and chlorinated oxidants and serve as novel markers of oxidative stress [1, 3]. Increased plasma levels of AOPPs have been found in aged populations [2]. Previous studies have reported that AOPPs can induce bone loss by breaking the balance between osteoblast-mediated bone formation and osteoclast-mediated bone resorption [5, 6, 9]. We have proven that AOPPs can induce massive ROS production in osteoblasts, decrease ΔΨm, damage mitochondria, and activate the mitochondria-dependent apoptosis pathway, eventually leading to cell apoptosis and osteopenia [5]. Based on our previous research, in this study, we found that upregulated mitophagy levels could protect osteoblasts from AOPP-induced apoptosis by clearing dysfunctional mitochondria and inhibiting excessive ROS production, therefore alleviating bone loss.

Our group and others have demonstrated that AOPPs can induce NOX-derived ROS generation and then cause ΔΨm depolarization in various cell types [5, 23]. Mitochondria are considered the main source of ROS in the cell, and ΔΨm depolarization results in the accumulation of damaged mitochondria and mtROS release, which play central roles in cell apoptosis [24]. However, previous studies did not discuss the effect of AOPPs on mtROS generation. Therefore, in this study, we investigated the relationship between AOPPs and mtROS production. We found that AOPP stimulation could induce mtROS generation in osteoblastic MC3T3-E1 cells, while the NOX inhibitor apocynin could inhibit mtROS production, which was supported by the RIRR mechanism. RIRR was first reported by Zorov et al. in the 2000s as a process involved in signal transduction between mitochondria, in which one cell (or organelle) triggers another cell (or organelle) to produce ROS [25]. Currently, RIRR is known to occur not only between mitochondria but also between NOXs and mitochondria, which links NOX-derived ROS and mtROS [12, 13]. In addition, we found that rapamycin lowered mtROS and total ROS levels, suggesting that a pro-mitophagy strategy may be effective in clearing AOPP-induced ROS accumulation in osteoblasts.

In this study, we demonstrated that AOPPs could induce PINK1/Parkin-mediated mitophagy in osteoblasts. Notably, even though both AOPPs and rapamycin could induce PINK1/Parkin-mediated mitophagy in osteoblasts, AOPP stimulation caused cell apoptosis, while rapamycin intervention protected osteoblasts from AOPP-induced cell death. The likely explanation may be that although both showing pro-mitophagic ability, they exert different effects on the upstream signaling pathway AMPK/mTOR/ULK1, which mediates mitophagy during cellular stress [26]. Specifically, AMPK plays an important role in maintaining mitochondrial homeostasis and mediates cellular stress signals by detecting a decrease in the intracellular ATP-to-ADP or ATP-to-AMP ratio [27] during ROS accumulation or energy shortage [26]. AMPK phosphorylation could activate mitophagy by directly inducing the phosphorylation of ULK1 or by suppressing the phosphorylation of mTOR [28]. mTOR is known to regulate growth signaling and to inhibit the phosphorylation of ULK1 under normal conditions [29, 30]. As our results showed, AOPPs promoted the phosphorylation of AMPK and inhibited mTOR phosphorylation, which indicated that the most important drivers of AOPP-induced mitophagy are massive ROS production and oxidative stress status. Rapamycin, a widely used mTOR inhibitor, only inhibited the phosphorylation of mTOR and had no effect on AMPK. This indicated that, in contrast to AOPPs, rapamycin-induced mitophagy in osteoblasts was independent of oxidative stress in our study. Therefore, we speculated that PINK1/Parkin-mediated mitophagy induced by AOPPs should be understood as a cellular self-protective mechanism during cell stress. However, the mitophagy level induced by AOPPs seemed inadequate to save osteoblasts from apoptosis as the AOPP concentration increased. Thus, the apoptotic rate could be ameliorated when rapamycin reinforced the ability to perform mitophagy.

The PINK1/Parkin-mediated mitophagy pathway is a well-known mitochondrial stress signaling pathway that mediates the specific ubiquitination and removal of damaged mitochondria by a selective autophagy mechanism [15, 16]. In recent years, the role of PINK1/Parkin-mediated mitophagy has been reported in numerous age-related diseases. For instance, in osteoarthritis rats, the expression level of PINK1/Parkin significantly decreased when compared with control, while curcumin exerted chondroprotective effects against osteoarthritis by promoting PINK1/Parkin-mediated mitophagy [31]. It was reported that Parkin-deficient mice accumulated damaged mitochondria in aging heart, while cardiac specific over-expression of Parkin delayed cardiac aging by enhancing mitophagy [32]. Furthermore, age-related hearing loss in C57BL/6 J mice is also associated with PINK1/Parkin-mediated mitophagy impairment [33].

In the present study, we found that AOPPs activated PINK1/Parkin-mediated mitophagy in osteoblasts in a dose-dependent manner. We believe that AOPP-induced oxidative stress and ΔΨm depolarization are the key factors in PINK1/Parkin-mediated mitophagy activation in this study. However, in mammals, a variety of functional mitochondrial receptors have been identified, such as BNIP3, BNIP3L/NIX, and FUNDC1, which contain an LC3 interaction region (LIR) motif that interacts with LC3, recruiting autophagosomes to mitochondria and inducing mitophagy [34–36]. BNIP3L/NIX-mediated mitophagy was first described in developing reticulocytes [37] and has been identified in neurons, renal cells, retinal ganglion cells, and several types of tumor cells [38–40]. Hypoxia has been considered a canonical stress factor that induces BNIP3L-mediated mitophagy [34]. FUNDC1 is an outer mitochondrial membrane protein that contains LIR and was identified as a mitophagy receptor by Liu et al. in 2012 [36]. During hypoxia, FUNDC1 dephosphorylates and activates mitophagy by binding directly with LC3 [36, 41]. Considering that hypoxia may occur secondary to ROS accumulation [42], it is possible that these receptor-mediated mitophagy processes might also be activated during AOPP stimulation. However, we still consider PINK1/Parkin-mediated mitophagy to play a leading role in this study.

Mitochondria are key players in the intrinsic apoptosis pathway [43, 44]. The interplay between mitophagy and apoptosis has been well studied in recent years. The activation of PINK1 signaling was reported to prevent cell death. PINK1 phosphorylates BCL-xL and prevents pro-apoptotic cleavage [45]. In addition, PINK1 phosphorylates the BH3-only protein, thereby regulating BAD and raising the threshold of apoptosis [46]. Parkin activation also prevents cell death. It was reported that Parkin could directly inhibit apoptosis by ubiquitinating the BH3-binding site of BAK and the N-terminus of cytosolic BAX [47]. To the best of our knowledge, mitophagy plays an important role in promoting osteoblast survival and relieving bone loss. Sun et al. reported that estrogen improved osteoblast cell proliferation by promoting mitophagy GPR30-ERK1/2 signaling in vitro [48]. Yang et al. showed that resveratrol prevented osteoblast dysfunction and osteoporosis in dexamethasone-treated rats by enhancing mitophagy, mediated by its effect on the SIRT1 and PI3K/AKT/mTOR signaling pathways [19]. In this study, by in vivo and in vitro experiments, we found that rapamycin further upregulated PINK1/Parkin-mediated mitophagy in mice receiving chronic AOPP loading, inhibited osteoblast apoptosis, partially restored bone volume, and improved trabecular bone quality.

Rapamycin is a canonical mTOR signaling inhibitor that has been widely studied in numerous bone metabolism-related studies with diverse conclusions. In some natural degenerative or pathological conditions, such as aging, inflammation, trauma, and certain gene-deficient models, rapamycin showed a pro-osteogenic effect. For instance, rapamycin could promote osteogenesis in an LPS-induced inflammatory environment [49], promote fracture healing [50], improve bone mass in elderly rats [51], inhibit bone loss in an OVX rodent animal model [52], and reverse the bone loss caused by FBN1 gene knockout [29]. In contrast, in adolescent animal models or in the process of cell differentiation, suppression of mTOR could result in retarded bone growth, which was shown as delayed growth plate formation or bone development with arrested cell differentiation [53]. Notably, previous studies reported that the bone mass of adult rats did not significantly change after 4 weeks of rapamycin administration [54]. In this study, rapamycin administration for 16 weeks had no significant effect on the bone mass of adult mice compared with the control group, which indicated that rapamycin did not markedly influence bone metabolism in normal mature animal models. Under pathological conditions, such as chronic stimulation with AOPPs, rapamycin intervention significantly restored bone mass.

In conclusion, by in vivo and in vitro studies, we found that PINK1/Parkin-mediated mitophagy could inhibit AOPP-induced osteoblast apoptosis by eliminating damaged mitochondria and scavenging ROS, thus inhibiting osteopenia and bone microstructural destruction. Therapeutic strategies aimed at upregulating mitophagy may preserve the mitochondrial function of osteoblasts against osteoporosis.

## Materials and methods

### AOPP preparation and determination

The preparation of AOPPs has been described previously [5, 6]. Briefly, mouse serum albumin (MSA, Sigma, St. Louis, MO, USA) solution (20 mg/ml) was incubated with 40 mM hypochlorous acid (Fluke, Buchs, Switzerland) at room temperature in phosphate-buffered saline (PBS, pH = 7.4) for 30 min. To remove the free hypochlorous acid, the prepared samples were dialyzed against PBS at 4 °C for 12 h. Finally, all samples were passed through a Detoxin-Gel™ Endotoxin Removing Gel (Pierce, Rockford, IL) to remove contaminated endotoxin. The endotoxin levels in AOPPs–MSA and unmodified MSA were measured by Limulus Amoebocyte Lysate kit (Sigma, St Louis, MO) and were found to be below 0.05 ng/mg. The concentration of prepared AOPP was detected as previously described [5, 6].

### Cell culture

Murine osteoblastic MC3T3-E1 cells were purchased from the Committee of Type Culture Collection (Chinese Academy of Sciences, Beijing, China). All in vitro studies were performed using passages 5–10. In this study, MC3T3-E1 cells were seeded in 25 cm^2^ flat-bottom cell culture flasks and supplemented with α-MEM (Gibco, Life Technologies, California, USA) containing 10% fetal bovine serum (Gibco, Life Technologies, California, USA) in a humidified atmosphere with 5% CO_2_ at 37 °C. In general, the cells were subcultured when they reached a subconfluent state after 3 days of culturing.

### Cell stimulation and reagents usage

MC3T3-E1 cells were incubated with increasing concentrations (0–200 μg/ml) of AOPPs for 24 h, in order to explore how AOPPs affect mitophagy level in this process. As for the reagents usage, MC3T3-E1 cells were pre-treated with rapamycin (100 nM), 3-MA (5 mM) or bafilomycin A1 (0.5 μM) for 2 h, and then co-stimulated with AOPPs (200 μg/ml) for 24 h. The concentration of MitoTEMPO in this study was 50 μM, and for apocynin, the concentration was 100 μM. Considering starvation itself may also induce mitophagy, the cells were not starved in the present study.

### Determination of cell apoptosis in vivo or in vitro

An Annexin V-FITC/PI double-staining kit (KeyGen Biotech, China) was used to detect the apoptosis rate in vitro. In brief, the collected cells were washed twice using 4 °C PBS and then resuspended in 500 μl of binding buffer. Next, 5 μl of FITC and 5 μl of PI were added to the buffer and incubated with MC3T3-E1 cells in the dark at room temperature for 15 min. Finally, all the collected cells were analyzed by FACSCalibur flow cytometer (Becton Dickinson, USA). Annexin V-FITC-positive/PI-negative and Annexin V-FITC-positive/PI-positive cells were considered to be apoptotic cells.

A TUNEL staining assay was used to explore apoptotic osteoblasts in the tibia bone tissue of C57BL/6 mice. Briefly, all tibias were removed, fixed in 4% paraformaldehyde, decalcified with 10% EDTA for 3 weeks, embedded in paraffin, and sliced into 5-μm-thick transverse sections following the standard method. Apoptotic cells in bone tissue were assessed with the deoxynucleotidyl transferase-mediated nick end labeling assay (TUNEL assay, Roche, Mannheim, Germany). Apoptotic osteoblasts were visualized under ZEISS LSM 880 (Carl Zeiss, Germany).

### Determination of ROS generation

Intracellular ROS were detected by probe 2’,7’-dichlorofluorescein diacetate (DCFH-DA, Sigma, St. Louis, MO, USA). In brief, MC3T3-E1 cells were incubated with 10 μM DCFH-DA for 30 min at 37 °C. Fluorescence intensity (Ex/Em = 488/525 nm) was measured on SpectraMax M5 system (Molecular Devices, California, USA).

Mitochondrial ROS were detected by MitoSOX (Invitrogen, California, USA) fluorescent probe. In brief, 5 µM MitoSox was added 40 min prior to analysis at 37 °C. Fluorescence intensity (Ex/Em = 510/580 nm) was measured on SpectraMax M5 system (Molecular Devices, California, USA).

### Cell immunofluorescence

MC3T3-E1 cells were seeded in confocal dishes with different treatments, fixed with paraformaldehyde, and permeabilized with 0.2% Triton-X. After blocking with 5% BSA, cells were incubated with rabbit anti-TOM20 (CST, #42406) or rabbit anti-HSP60 (CST, #4870) overnight at 4 °C. After washing and incubating with FITC-labeled goat anti-rabbit IgG (Beyotime, China) or Cy3-labeled goat anti-rabbit IgG (Beyotime, China). For immunofluorescence co-localization, cells with different treatments were fixed, permeabilized and blocked as mentioned above, and incubated with rabbit anti-LAMP1 (abcam, ab208943) and mouse anti-COX IV (abcam, ab33985) overnight at 4 °C, and then incubated with Cy3-labeled goat anti-rabbit IgG (Beyotime, China) and FITC-labeled goat anti-mouse IgG (Beyotime, China). All of the cells were stained by DAPI (Abcam,Cambridge,UK), and observed by ZEISS LSM 880 (Carl Zeiss, Germany).

### LysoTracker red and MitoTracker green staining

LysoTracker Red (Beyotime, China) and MitoTracker Green (Beyotime, China) were used to detect lysosomes and mitochondria, respectively. In brief, cells were pre-incubated with LysoTracker Red (50 nM) and MitoTracker Green (100 nM) for 30 min at 37 °C in the dark, washed three times with PBS, and observed with confocal microscope (Olympus, Tokyo, Japan).

### Transmission electron microscope

MC3T3-E1 cells were digested and collected, and the cells were suspended and fixed with 2.5% glutaraldehyde in PBS (pH = 7.4) at 4 °C for 2 h. After washing three times with PBS, the cells were treated with conventional dehydration, osmosis, embedding, sectioning and staining. The ultrastructure of the cells was observed under Hitachi H7700 electron microscope.

### Determination of mitochondrial membrane potential

The change in mitochondrial membrane potential was detected by fluorescent dye JC-1 (KeyGen Biotech, China). Briefly, cells seeded into confocal dishes were incubated with a mixture of 1 μM JC-1 staining fluid and 500 μl of incubation buffer in the dark at 37 °C for 30 min, washed twice with incubation buffer, and observed with confocal microscope (Olympus, Tokyo, Japan).

### Western blot

Cultured cells were washed three times with 4 °C PBS and then lysed with RIPA lysis buffer. The supernatant was centrifuged at 4 °C and 13800×g for 30 min to collect the protein. BCA Protein Assay Kit (Thermo, Life Technologies, California, USA) was used to determine the protein concentration. The samples were separated by SDS-polyacrylamide gel electrophoresis (PAGE) using 6–15% acrylamide gels and then transferred to polyvinylidene fluoride (PVDF) membranes (Millipore, Billerica, MA, USA). After incubation with primary and secondary antibodies, the protein bands were detected with chemiluminescence detection reagents (Millipore, Billerica, MA, USA). The following antibodies were used. Apoptosis Antibody Sampler Kit (#9930), Autophagy Antibody Sampler Kit (#4445), rabbit anti-BAX (#2772), rabbit anti-cytochrome c (#4272), rabbit anti-Parkin (#32833), rabbit anti-GAPDH (#2118), rabbit anti-ULK1 (#8054), rabbit anti-p-ULK1 (#5869), rabbit anti-AMPK (#5832) and rabbit anti-p-AMPK (#2535) were all from Cell Signaling Technology (CST, Beverly, MA, USA). Rabbit anti-PINK1 (ab23707), rabbit anti-mTOR (ab134903), rabbit anti-p-mTOR (ab109268) were from Abcam (Abcam, Cambridge, UK). Goat anti-rabbit IgG H&L (HRP) (ab205718) secondary antibodies were from Abcam (Abcam, Cambridge, UK). The integrated density of all the protein bands was analyzed with ImageJ software.

### Lentiviral vector infection

The lentiviral vector was purchased from Genomeditech (Shanghai, China). The sequence for specifically targeting mouse PINK1 was CCTGGCTGACTATCCTGATAT (5’–3’); the sequence for the nontargeting lentiviral vector was TTCTCCGAACGTGTCACGT (5’–3’). For lentiviral transfection, MC3T3-E1 cells were grown to 30% confluence and then transfected with the lentiviral RNAi vector targeting PINK1 or the nontargeting lentiviral vector for 72 h.

### Animal studies

All animal experiments in this study were approved by the Committee on Animal Experimentation and the Laboratory Animal Care and Use Committee of Southern Medical University. All the mice were randomly divided into five groups containing eight mice per group and received the following treatments: group 1, daily intraperitoneal injection of vehicle (PBS, pH = 7.4); group 2, daily intraperitoneal injection of unmodified MSA (50 mg/kg); group 3, daily intraperitoneal injection of AOPPs (50 mg/kg); group 4, daily intraperitoneal injection of AOPPs (50 mg/kg) together with rapamycin (1 mg/kg); and group 5, intraperitoneal injection of rapamycin (1 mg/kg). At the end of 16 weeks, the animals were sacrificed. Blood samples were collected by abdominal aorta puncture and centrifuged for plasma. The tibias were collected and fixed in 4% paraformaldehyde for subsequent analysis.

### Plasma AOPP and bone-specific alkaline phosphatase (B-ALP) concentration

The method of determining AOPP levels in plasma was the same as the method used for AOPP content determination in the AOPP–MSA compound. The bone-specific alkaline phosphatase (B-ALP) level in the plasma was determined by ELISA kit (CUSABIO, China) according to the manufacturer’s protocol.

### Micro-CT analysis

Microcomputed tomographic (micro-CT) examination was carried out with Scanco μCT-80 CT scanner system. All the samples were scanned at a nominal resolution (pixels) of 12 μm with a source voltage of 55 kV. The volume of interest (VOI) was defined as starting approximately 1.2 mm from the growth plate extending for an additional 1.6 mm of the proximal tibia. The bone mineral density (BMD) of the trabeculae of tibias and parameters of the trabecular bone microstructure, such as bone volume over total volume (BV/TV), trabecular thickness (Tb. Th), trabecular number (Tb. N), trabecular spacing (Tb. Sp), and structure model index (SMI) were calculated with the μCT’s software.

### Immunohistochemical and immunofluorescent staining of bone tissue

Paraffin-embedded bone tissue was sliced into 5-μm-thick transverse sections. After the process mentioned above, slices were incubated with primary antibodies against BAX and cleaved PARP at 4 °C overnight. The immunostaining was examined with Leica DM5000 B (Leica, Germany). For immunofluorescent staining, the sliced bone tissue was incubated with PINK1 conjugated to PI or Parkin conjugated to FITC. DAPI was used to label the nucleus. The immunofluorescent staining was visualized under ZEISS LSM 880 (Carl Zeiss, Germany).

### Statistical analysis

All the experiments in this study were repeated at least three times. Continuous variables are presented as the mean±standard deviation (SD). One-way ANOVA was used to detect differences among groups. A two-tailed *p* value of <0.05 was considered statistically significant. Statistical analysis were conducted with SPSS 20.0 software (SPSS Inc., Chicago, IL).

## Supplementary information

Supplementary Material Figure

Checklist

Original Data File

## Data Availability

The data that support the findings of this study are available from the corresponding author upon reasonable request.
