# Supplementary material for: PINK1/Parkin-mediated mitophagy inhibits osteoblast apoptosis induced by advanced oxidation protein products
Source: Cell Death Dis. 2023 Feb 7;14(2):88. doi: 10.1038/s41419-023-05595-5 (PMC9905061; doi:10.1038/s41419-023-05595-5)
Supplement: Supplementary file 1 — Supplementary Material Figure [file 41419_2023_5595_MOESM1_ESM.pdf]

Supplementary Material

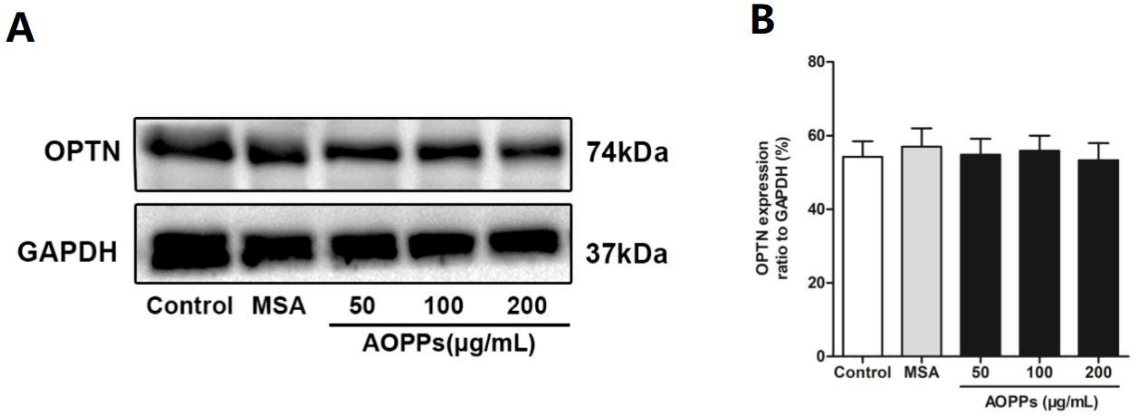

Supplementary Material Fig.1 A-B: Optineurin (OPTN) expression levels in AOPPs stimulated (0-200 μg/ml, 24 h) MC3T3-E1 cells. No significant difference was detected among each group.
