# Supplementary figures and images for: PINK1/Parkin-mediated mitophagy inhibits osteoblast apoptosis induced by advanced oxidation protein products
Source: Cell Death Dis. 2023 Feb 7;14(2):88. doi: 10.1038/s41419-023-05595-5 (PMC9905061; doi:10.1038/s41419-023-05595-5)

# Figure2

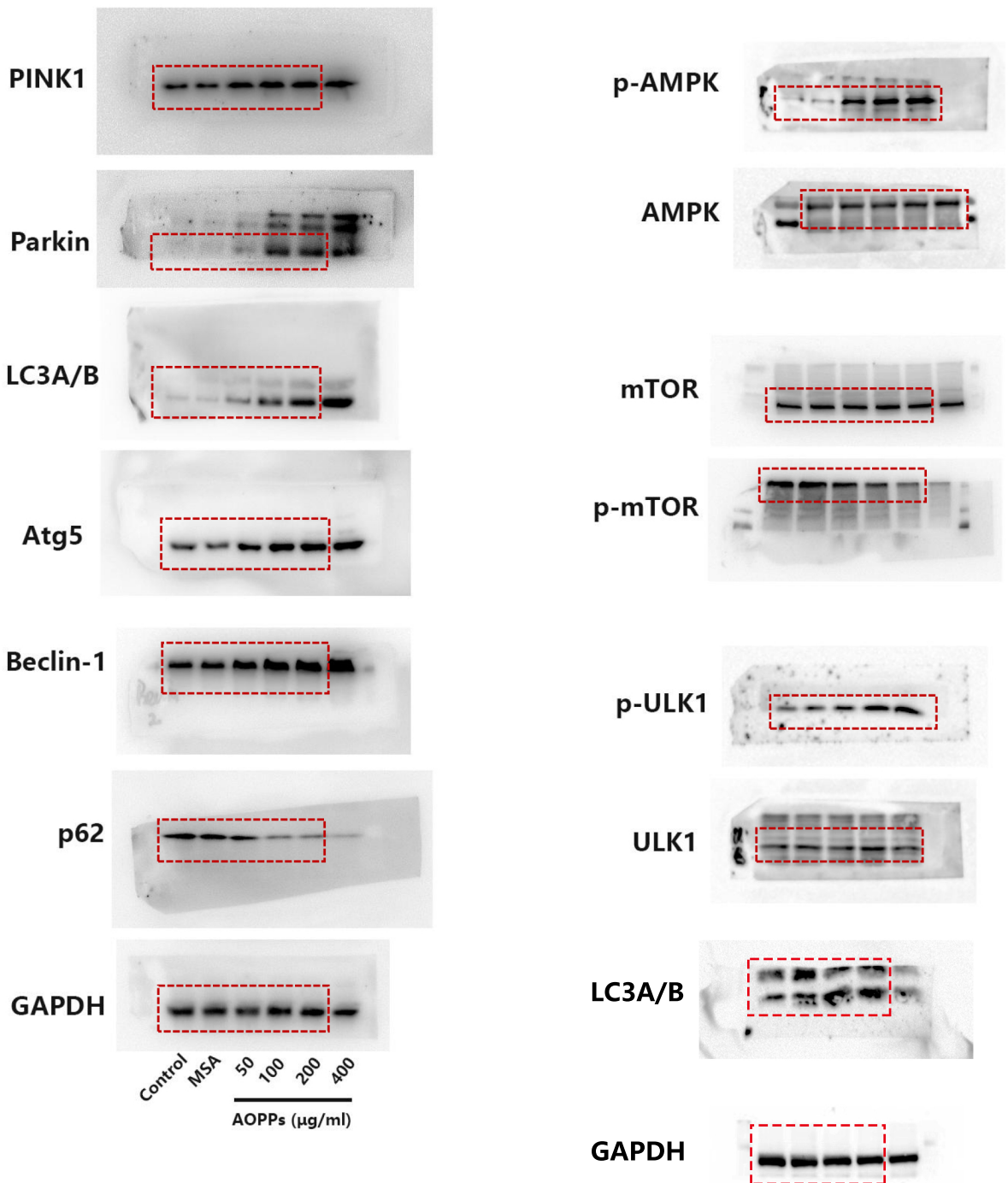

# Figure3

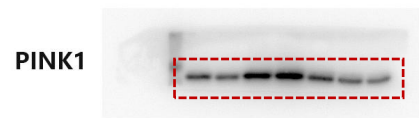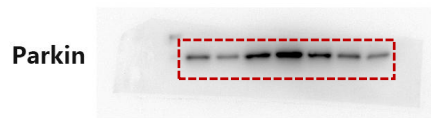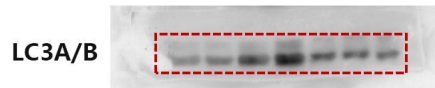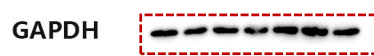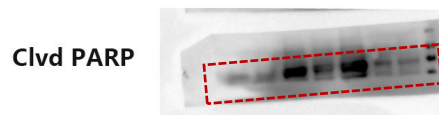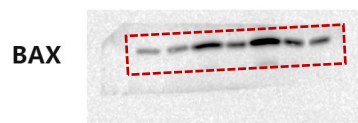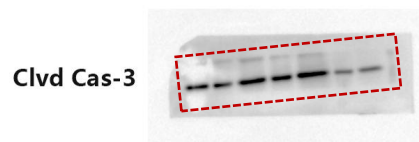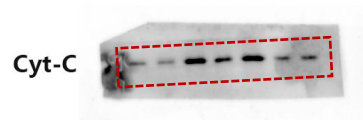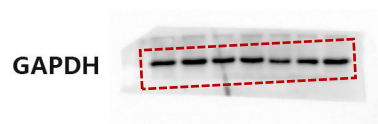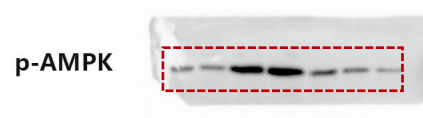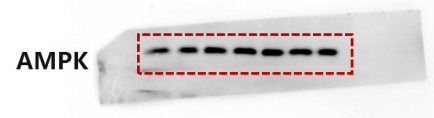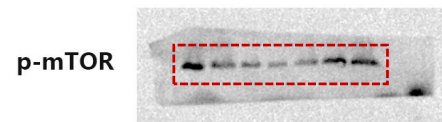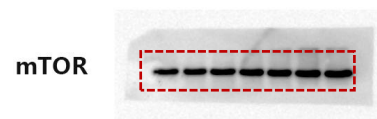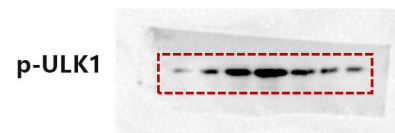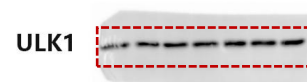

**Figure4**

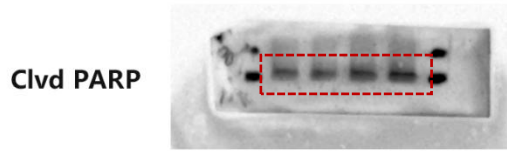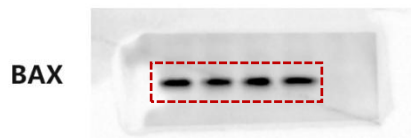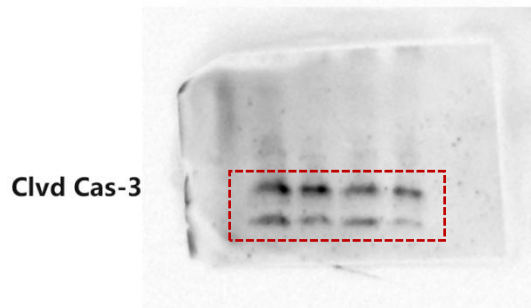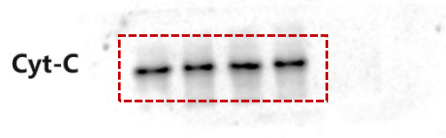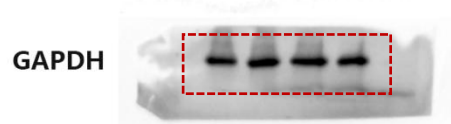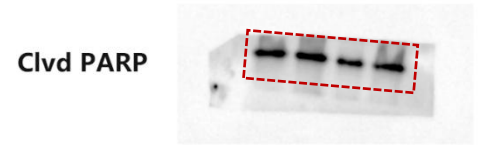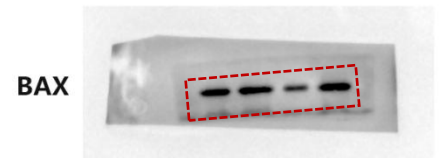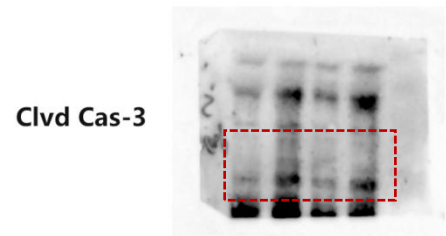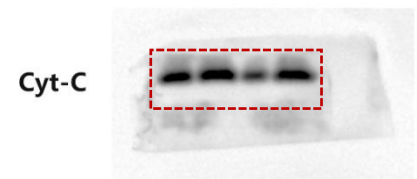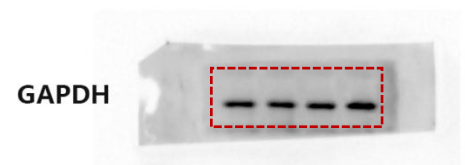

**Figure6 & Figure7**

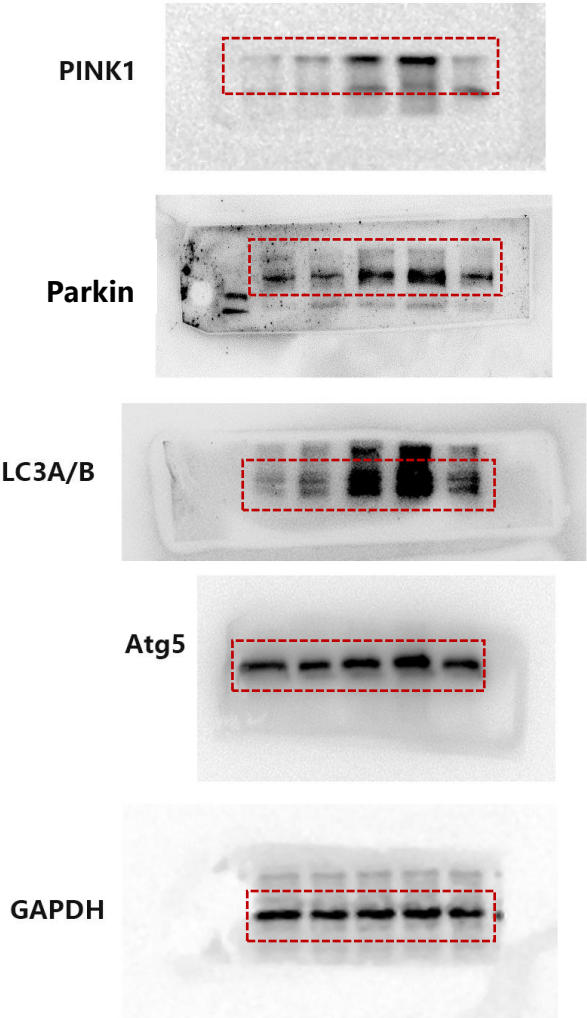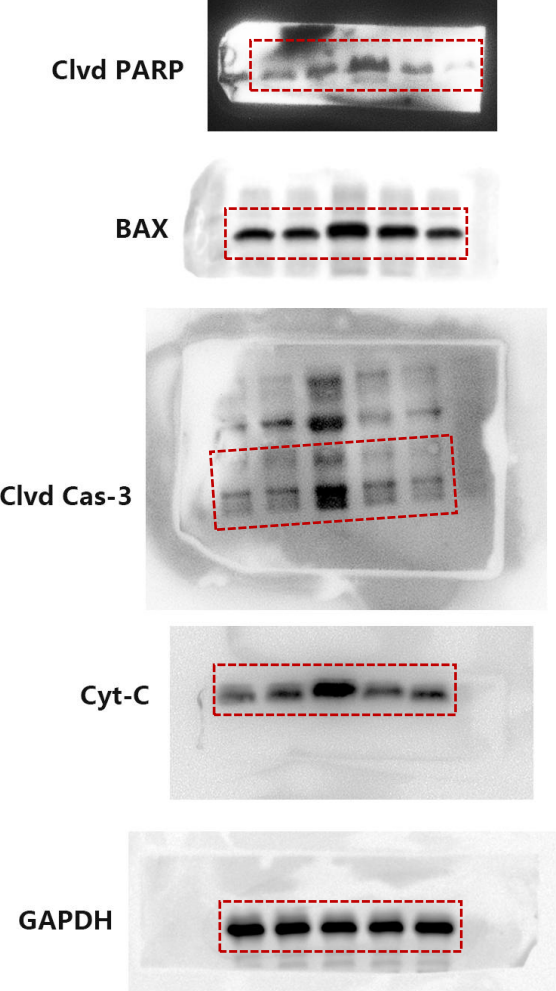

Supplement: Supplementary file 3 — Original Data File [file 41419_2023_5595_MOESM3_ESM.pdf]
